# Novel Insights into the Genetics and Pathophysiology of Adrenocortical Tumors

**DOI:** 10.3389/fendo.2015.00096

**Published:** 2015-06-09

**Authors:** Ludivine Drougat, Hanin Omeiri, Lucile Lefèvre, Bruno Ragazzon

**Affiliations:** ^1^U1016, INSERM, Institut Cochin, Paris, France; ^2^UMR8104, CNRS, Paris, France; ^3^Université Paris Descartes, Sorbonne Paris Cité, Paris, France

**Keywords:** adrenocortical adenoma, hyperplasia, adrenocortical carcinoma, PRKACA, ARMC5, ZNRF3

## Abstract

Adrenocortical tumors (ACTs) are typically unilateral and can be classified as benign adrenocortical adenomas (ACAs) or malignant adrenocortical cancers (ACCs). In rare cases, tumors may occur in both adrenal glands as micronodular hyperplasia (primary pigmented nodular adrenal dysplasia) or as macronodular hyperplasia (primary bilateral macronodular adrenal hyperplasia, PBMAH). The study of certain tumor predisposition syndromes has improved our understanding of sporadic ACTs. Most ACAs are associated with abnormalities of the cAMP signaling pathway, whereas most ACCs are linked to alterations in IGF2, TP53, or the Wnt/βcatenin pathways. Over the past year, single-nucleotide polymorphism array technology and next-generation sequencing have identified novel genetic alterations in ACTs that shed new light on the molecular mechanisms of oncogenesis. Among these are somatic mutations of PKA catalytic subunit alpha gene (*PRKACA*) in ACA, germline, and somatic mutations of armadillo repeat containing 5 gene (*ARMC5*) in primary bilateral macronodular adrenal hyperplasia and somatic alterations of the E3 ubiquitin ligase gene *ZNRF3* in ACC. This review focuses on the recent discoveries and their diagnostic, prognostic, and therapeutic implications.

## Introduction

The pathogenic mechanisms underlying adrenocortical tumors (ACTs) are complex and heterogeneous. The most common ACT is benign, unilateral, non-secreting (adrenocortical adenomas, ACAs-NS), and often discovered incidentally. ACTs exist in the bilateral form but are much less frequent. The symptoms due to ACT are caused by steroid excess (Cushing’s syndrome) in the case of secreting benign tumors. The aggressive and deadly forms of ACT are adrenocortical cancers (ACCs) but have an overall low incidence of appearance. The clinical consequences of ACC can be due to steroid oversecretion, tumor growth, or metastasis. ACCs are rare and show heterogeneity in malignancy, in levels of hormone secretion, and in tumor progression. It is also difficult to predict evolution and prognosis although these cancers are globally associated to poor outcome.

Till now, the majority of genetic and molecular alterations of benign tumors has been closely linked to abnormalities in the cAMP signaling pathway. Somatic and germline mutations were identified in actors of the cAMP pathway as the *PRKAR1A* gene (*regulatory subunit of the cAMP-dependent protein kinase A*) (1, 2), *GNAS* gene (α *subunit of the stimulatory G protein*) (3), and the *PDE11A*/*8B* genes (*cAMP-degrading phosphodiesterase 11A and 8B*, respectively) (4, 5). Other alterations modulating the cAMP/PKA pathway activity that stimulates steroidogenesis are present in ACA. For example, ectopic expression of the gastric inhibitory polypeptide receptor (GIPR) in the human adrenal gland causes significant hypercortisolemia after meal ingestion and leads to Cushing’s syndrome (6, 7). Ectopic expression of other receptors belonging to binding G protein-coupled receptors classes such as vasopressin, serotonin, and catecholamine receptors have been described in the bilateral hyperplasias of the adrenal cortex and cortisol-secreting adenomas (ACA-S) (8, 9). In contrast to ACA, ACCs have been related to alterations in various pathways such as IGF2, TP53, or Wnt/βcatenin. Initially, progress in identifying genes involved in sporadic ACT came mainly from the study of rare familial cases (10–12): *TP53* tumor suppressor gene and its predisposition’s locus on chromosome 17p13.1 involved in Li–Fraumeni syndrome; the imprinted gene encoding the insulin-like growth factor IGF2, located on chromosome 11p15.5 and associated with Beckwith–Wiedemann syndrome, germline *PRKAR1A* mutations identified in Carney complex. Moreover, somatic mutations in the *CTNNB1* gene have been reported in both benign and malignant ACTs (13). However, alterations in these several genes are identified only in subgroups of ACA and ACC. Over the last 5 years, the development of high-throughput sequencing has revealed several frequent alterations in genes not previously described, underlying new insights in the pathogenesis of benign and malignant forms of ACT. For example, a hotspot somatic mutation in the PKA catalytic subunit alpha gene (*PRKACA*) has been identified in ACA (14), germline, and somatic mutations of armadillo repeat containing 5 gene (*ARMC5*) have been described in patients with primary bilateral macronodular adrenal hyperplasia (PBMAH) (15), and somatic alterations in the E3 ubiquitin ligase gene *ZNRF3* were recently identified in ACC (16). In this review, we aim to give an overview of recent advances in the genetics of ACT, focusing on the latest driver genes identified, and therefore improving our understanding of the pathophysiology of these tumors.

## Adrenocortical Adenomas

**Figure 1:**
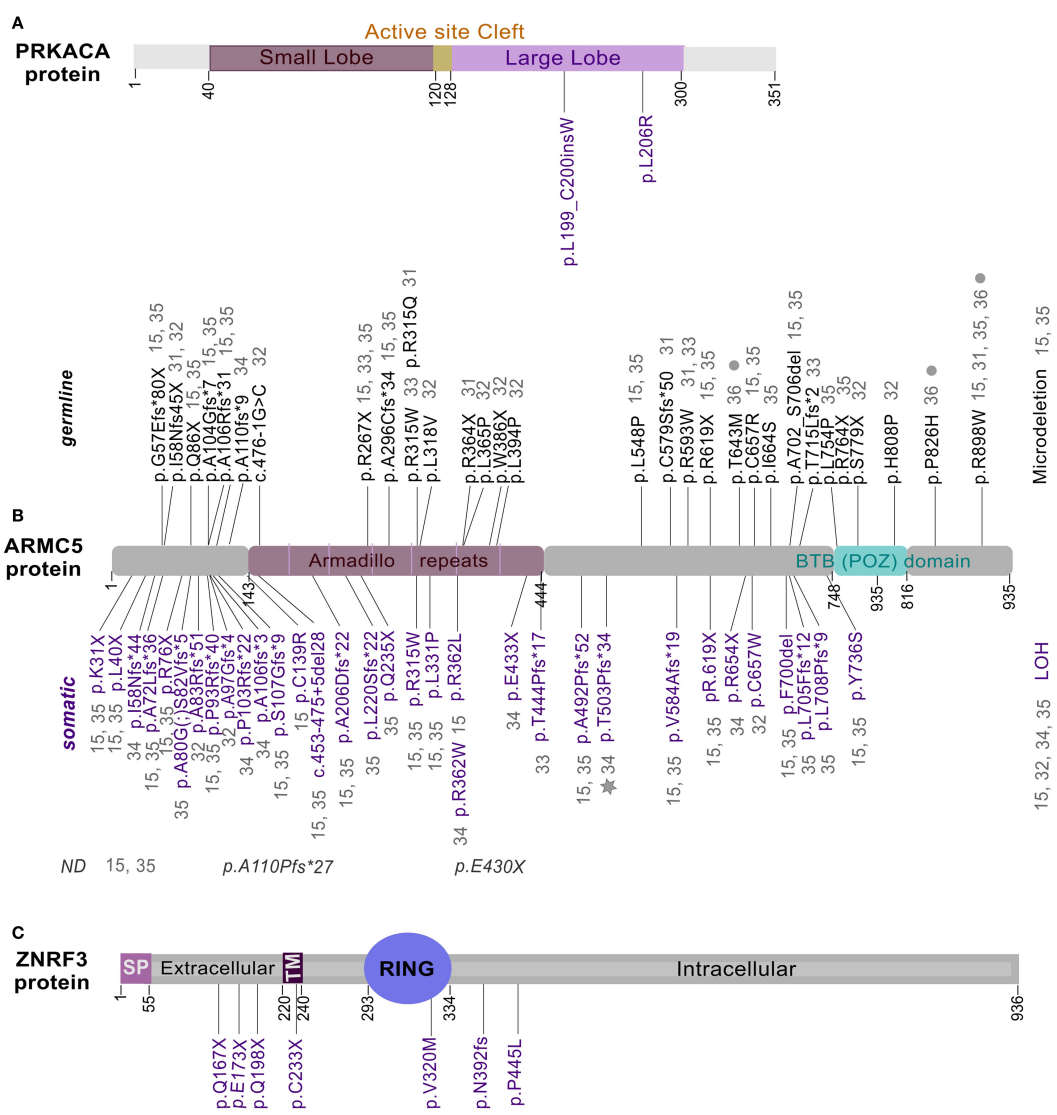
**Location of mutations in PRKACA, ARMC5, and ZNRF3**. Full length of PRKACA **(A)**, ARMC5 **(B)**, and ZNRF3 **(C)** proteins were represented with mutations identified in ACA-S (14, 21–24), PBMAH (15, 27–32), and ACC (16), respectively. Somatic mutations are shown in purple, while germline mutations in black. Gray star and filled gray circles represent mutations identified in a meningioma (31) and in patients with hyperaldosteronism (32), respectively. ND, germline or somatic status not determined.

**Figure 2:**
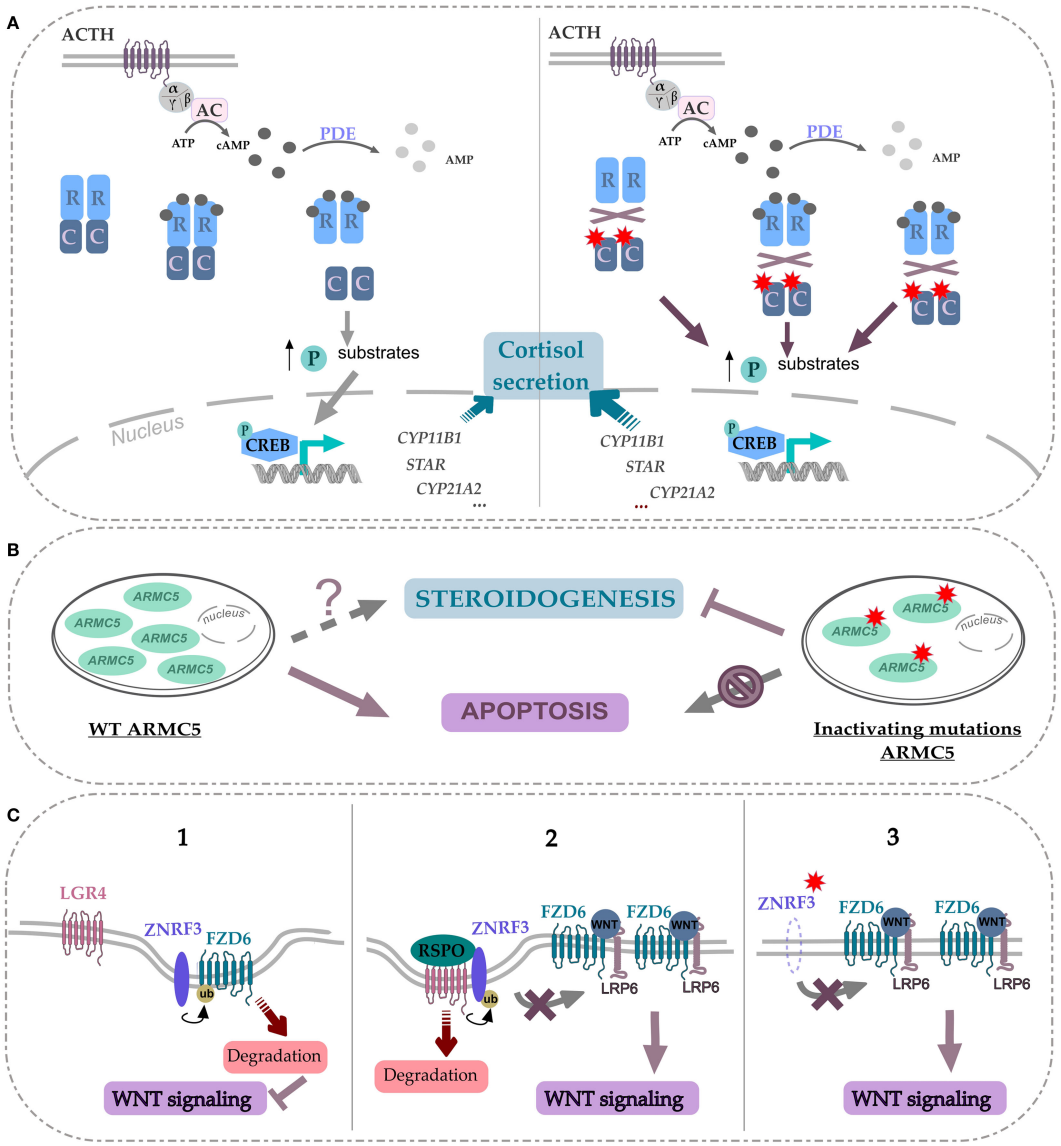
**Effects of recently identified drivers in signaling pathways involved in adrenal tumorigenesis**. **(A)** Alterations in the PKA pathway caused by PRKACA^p.L206R^ and PRKACA^Leu199_Cys200insTrp^ mutations in adrenocortical adenomas. Under normal conditions, the catalytic and regulatory subunits of PKA form a heterotetramer. In response to ACTH stimulation, the catalytic subunits are released from the complex in an active form. The free catalytic subunits phosphorylate their substrates and thereby increase the transcription of target genes. These target genes are mainly involved in the synthesis of cortisol and other steroids. The p.L206R and Leu199_Cys200insTrp mutations in the *PRKACA* gene alter its interaction with regulatory subunits of PKA leading to constitutive activation of PKAcα and increased steroidogenesis. **(B)** ARMC5, a new indirect or direct regulator of steroidogenic and apoptosis processes. ARMC5 inactivating mutations induce a decreased steroidogenic capacity and a protection against cell death. **(C)** ZNRF3 a negative feedback regulator of the Wnt/β-catenin signaling pathway. 1, In the absence of R-spondin (RPSO), ZNRF3 induces the poly-ubiquitination of Frizzled receptors (FZD) that promote their degradation. 2, In the presence of RSPO, ZNRF3 interacts with the complex RSPO/LGR4, which results in the turnover of ZNRF3 and thus the stability of FZD and the stimulation of Wnt/β-catenin signaling. 3, In ACC, ZNRF3 alterations can produce similar effects of RSPO stimulation leading to an activation of Wnt/β-catenin signaling. Red stars represent mutated or altered proteins.

Prior to the introduction of next-generation sequencing, mutations in some genes such as *GNAS* or *PRKAR1A* had been reported in ACA-S. Activating mutations of the *GNAS* alpha subunit (17) and *PRKAR1A*-inactivating mutations (18) promote the cAMP pathway activation. *CTNNB1*-activating mutations had been found in ACA-NS and ACA-S but their prevalence was higher among ACA-NS (13, 19, 20). However, these mutations accounted for only a subset of ACA. Recently, Beuschlein and collaborators identified a hotspot mutation in *PRKACA* gene through whole-exome sequencing in ACA-S (14). The somatic mutation, p.L206R/c.617A > G was present in more than one-third of the examined tumors. This result was confirmed by four other groups, which has reported the same recurrent mutation in the *PRKACA* gene (21–24). This mutation occurs in the C-terminus of the activation segment in the *p* + 1 loop of PRKACA protein (Figure 1A). This region is a specific binding site for the interaction between catalytic and regulatory subunits of PKA (25). The p.L206R point mutation results in the introduction of a voluminous and positively charged amino acid that inhibits the formation of stable complexes between subunits of PKA (23, 24, 26). This mutation prevents the interaction of the catalytic subunit of PKA with the regulatory subunit, resulting in an increased phosphorylation of substrates and finally, in an excessive steroidogenic activity (Figure 2A). The consequence of this lack of interaction has been shown for both RIA (*PRKAR1A*) and RIIB (*PRKAR2B*) regulatory subunits (26). L206R mutation of *PRKACA* in ACA-S was associated with more severe phenotypes (Cushing’s syndrome) (14). Another mutation in the *PRKACA* gene, Leu199_Cys200insTrp, identified only in one study, has the same effect on the stability of the PKA complex (14, 26) (Figures 1A and 2A).

Adrenal cortex and cortisol-secreting adenomas are characterized by a high occurrence of *PRKACA-*activating mutations. However, other mutations in *GNAS* and *CTNNB1* genes are found in some ACA-S without *PRKACA* mutations and are mutually exclusive (21, 23). The hotspot mutation in the *PRKACA* gene seems to be sufficient to alter the endocrine and proliferative systems in ACA-S and represents the main genetic risk factor associated with this type of tumor (14, 21–24).

## Primary Bilateral Macronodular Adrenal Hyperplasia

Primary bilateral macronodular adrenal hyperplasia described first in 1964 is a rare type of bilateral ACTs leading to adrenal Cushing’s syndrome (33). PBMAH are often revealed incidentally during radiological examinations or by the presence of overt Cushing’s syndrome. Both adrenal glands are enlarged massively with the presence of numerous macronodules. This adrenal disorder is usually diagnosed in patients aged between 40 and 60. In addition to ectopic expression of G protein-coupled receptors, it has been described in PBMAH an abnormal expression of paracrine factors (34–36). For instance, recently, an ACTH production by adrenocortical cells was reported in a large series of PBMAH, which can play a role in cortisol hypersecretion (36). Despite the fact that most cases of PBMAH appeared to be sporadic, some familial cases were reported, supporting the idea of a germline hereditary factor. Mutations or variants of some genes involved in the cAMP signaling pathway have been identified as in *GNAS*, *PDE11A*, and *PDE8B* genes but are only present in a limited fraction of PBMAH cases.

Combining single-nucleotide polymorphism (SNP) array and whole-genome sequencing, the first gene predisposing to PBMAH in adults has been recently identified (15). The most frequent somatic chromosome alteration in PBMAH was a loss of heterozygosity (LOH) at 16p and, the most frequent mutation identified was in *ARMC5* gene, located at 16p11.2. *ARMC5* alterations were detected in tumors obtained from 18 of 33 patients who had undergone surgery (55%). In all cases, both alleles of *ARMC5* carried alteration: one germline and the other somatic. For some cases with an *ARMC5* germline mutation, different nodules from one or both adrenal glands were analyzed. In each case, the same germline mutation was detected in all nodules and associated with a nodule-specific second somatic *ARMC5* alteration (LOH, nonsense or missense mutation). The discovery of *ARMC5* alterations establishes the first direct genetic link to PBMAH. The pattern of mutations suggests a “two-hit” model of a tumor suppressor gene, responsible for a hereditary predisposition syndrome. Subsequent studies confirm the recurrent mutation of *ARMC5* in family members with PBMAH (27–30). In these various studies, the percentage of *ARMC5* mutations reaches 25% in index cases of PBMAH. Recently, the high frequency of alterations in the *ARMC5* gene has been confirmed in a large cohort of 98 patients with PBMAH, including operated and non-operated patients (31). Up to now, these recent studies identified – in patients with PBMAH – in addition to LOH and a microdeletion, a total of 61 different mutations in *ARMC5*: 27 germinal, 30 somatic, two which have been identified at somatic and germline levels in different cases (p.R315 and p.R619X) and two without status available (Figure 1B). All these mutations can be found all along the protein in different domains. Two of the germline mutations are found in several index cases and in at least three studies suggesting a founder effect: p.R267X and p.R898W (15, 27–32).

The *ARMC5* encodes a protein of 935 amino acids and the peptide sequence reveals two distinctive domains: ARM domain in the N-terminal and a BTB/POZ in the C-terminal (Bric-a-Brac, Tramtrack, Broad-complex/Pox virus, and Zinc finger) (Figure 1B). These domains are highly conserved through evolution and have been shown to be involved in mediating protein-protein interactions, but targeted proteins recognition by these domains is not understood yet. The mechanism of ARMC5 action is unknown because no study has ever been focused on its biological function, and no diseases have been associated with the *ARMC5* gene until now. Recent functional study on *ARMC5* gene, performed in the human adrenocortical cells H295R, showed that *ARMC5* gene silencing alters the expression of genes involved in steroidogenesis leading to a global decreased of cortisol secretion (15) (Figure 2B). These data are consistent with previous expression-profile studies (37, 38). It is therefore likely that, despite the reduced secretory capacity of each cell, the overall production of cortisol was increased because of the large adrenal mass. All data describing *ARMC5* mutations show that patients suffering from PBMAH have a phenotype more severe than patients without *ARMC5* mutation (15, 31). Patients with ARMC5 mutations present with larger tumor volumes, increased numbers of tumor nodules, and more severe hypercortisolism (31). Recently, *ARMC5* mutations have been associated with another steroid hypersecretion. Indeed, six patients of 56 (10.7%) with primary hyperaldosteronism had germline mutations in the *ARMC5* gene. Among these six patients, two suffered from PBMAH (32).

The genomic and functional data indicate that *ARMC5* has a role of tumor suppressor gene because two inactivating mutations seem necessary to develop PBMAH and human cells (H295R and HeLa) transfected with non-mutated ARMC5 resulted in cell death (Figure 2B). In contrast, this effect was not observed with missense mutations. This suggests that ARMC5 plays a significant role in cell apoptosis (15, 31).

Bilateral adrenalectomy is considered as the single treatment of choice for PBMAH, the finding of *ARMC5* gene is promising for the discovery of new therapeutic perspectives. Interestingly, a somatic mutation in *ARMC5* gene has also been found in a meningioma in patients with an *ARMC5* germline mutation and a PBMAH (30). These data suggest that genetic alterations of the *ARMC5* gene may cause the development of different associated tumors with PBMAH. With the recent advances in the genetic methods, it is possible to imagine that future studies will reveal cases with *ARMC5* mutations in other types of tumors without PBMAH. Now, it is necessary to better know the functional role of the ARMC5 protein in order to understand the impact of these mutations on the initiation and/or development of PBMAH.

Mutations in the *DOT1L* (DOT1-like histone H3K79 methyltransferase) and *HDAC9* (histone deacetylase 9) genes have also been found in patients with PBMAH. Unlike *ARMC5* mutations, their frequency is lower and appeared only in two and one cases, respectively. These new mutations seem to define a little subgroup of PBMAH without *ARMC5* mutations (21). DOT1L and HDAC9 are methyltransferase and histone deacetylase, respectively; these two nuclear proteins are involved in the transcriptional regulation. Further investigations will help to delineate the importance of these three genes in the adrenal function. In regard to the high frequency (20%) of mutations in *ARMC5* gene in all index cases analyzed, its systematic genetic screening appears to be important for patients with PBMAH or Cushing syndrome. This screening can be used for early detection of PBMAH in family members with no clinical evidence.

## Adrenocortical Cancer

ACC is a rare and highly aggressive endocrine tumor that affects one to two persons per 1 million of the population per year (39). The prognosis of ACC is very poor, with a 5-year survival rate under 35% in most series (40–43). Currently, surgery is the only curative therapy available. Medical treatments, including the adrenolytic drug mitotane and cytotoxic chemotherapy, show only limited therapeutic potential (44). The rarity of ACC is a limiting factor in the progress to understand the pathophysiology of this tumor. Up to now, somatic inactivating mutations of the tumor suppressor gene *TP53* and activating mutations of the proto-oncogene β*-catenin* (*CTNNB1*) were the most frequent mutations identified in ACC (13, 42, 45, 46).

Recently, a cohort of 122 ACC, from the European Network for the Study of Adrenal Tumors (ENSAT), was analyzed by SNP array. Fifty-five of these 122 ACC have also been analyzed by a combination of other genomic approaches, including exome sequencing, DNA methylation, mRNA expression arrays, and miRNA sequencing. Candidate driver genes were validated by targeted sequencing in all tumors. This work confirmed recurrent alterations in the known drivers *CTNNB1* and *TP53* and revealed new genes not previously reported to be altered in ACC. Strikingly, *ZNRF3* (Zinc and ring finger protein 3) was the most frequently altered gene (21%). In a majority of cases, homozygous deletions of *ZNRF3* were observed but few somatic inactivating mutations and two missense mutations were also identified (16) (Figure 1C).

*ZNRF3* and its homolog *RNF43* (ring finger protein 43) encode proteins with E3 ubiquitin ligase activity that have recently been described as cell-surface transmembrane E3 ubiquitin ligases, acting as negative feedback regulators of Wnt/β-catenin signaling. ZNRF3 and RNF43 contain a signal peptide, an extracellular domain for R-spondin (RSPO)-binding, a single transmenbrane helix, a cytoplasmic really interesting new gene (RING) finger domain, and a C-terminal tail. It has been demonstrated that ZNRF3/RNF43 are associated with the Wnt receptors (Frizzled, FZD), which results in a multi-ubiquitination of lysines in the intracellular domain of FZD and then their internalization and degradation in lysosomes (47, 48). RSPO are secreted proteins known to potentiate the Wnt signaling. Various membrane proteins have been reported to bind RSPO, including FZD and LRP6, LGR4/5/6, Kremen, Syndecan, and ZNRF3/RNF43 (49). Several models of RSPO signaling have been proposed. Recently, published data indicate that the ZNRF3/RNF43-mediated membrane clearance of FZD is reversed upon addition of RSPO (47, 49, 50). Once bound to its receptor (LGR5), RSPO are believed to decoy ZNRF3, thus permitting strong β-catenin signaling (Figure 2C).

It has been shown that ZNRF3 protein expression is down regulated in gastric adenocarcinoma tissues compared with adjacent normal gastric tissues (51). Recurrent deletion of three regions in chromosome 22 was identified in osteoblastoma, one of these regions contains *ZNRF3* (52). Moreover, the deletion of *ZNRF3* and *RNF43* in the intestinal epithelium in mouse induces the development of adenoma with an increased nuclear β-catenin and an increased expression of Wnt/β-catenin target genes (48).

Interestingly alterations of *ZNRF3* and *CTNNB1* are completely exclusive in ACC (16), suggesting that *ZNRF3* alterations might play a crucial role in tumorigenesis by activating also the Wnt/β-catenin signaling pathway. Taken together, 37% of ACC samples harbored an alteration affecting the Wnt pathway. These data strongly suggest that in ACC, *ZNRF3* is a tumor suppressor gene related to the Wnt pathway. ACC with altered *ZNRF3* showed transcriptional activation of β-catenin targets, but this activation was weaker than in *CTNNB1*-mutated tumors (16). However, till now, ACCs are the cancers described with the most frequent *ZNRF3* alterations, suggesting a specific mechanism of tumorigenesis into the adrenal cortex tissue. Future functional studies are needed to investigate its role in adrenocortical cells.

## Conclusion

Analyses of inherited syndromes associated with an increased risk adrenocortical tumorigenesis, coupled with recent advances in sequencing technology, have improved our understanding of ACT. Recent advances in genomic tools, especially sequencing technologies, have yielded new findings in three types of ACT. Alterations in genes not previously reported were identified: somatic mutations of *PRKACA gene* in ACA, germline and somatic mutations of *ARMC5* gene in PBMAH, and somatic alterations of *ZNRF3* gene in ACC.

It would be worth pursuing functional studies on these genes in order to understand the impact of these alterations on the initiation and/or development of ACT. The identification of signaling pathways playing a major role in ACT development would help to develop new targeted therapies, which are dramatically needed for the management of patients harboring these tumors, especially for ACC.

## Conflict of Interest Statement

The authors declare that the research was conducted in the absence of any commercial or financial relationships that could be construed as a potential conflict of interest. The Associate Editor Pierre Val declares that, despite having collaborated with author Bruno Ragazzon, the review process was handled objectively and no conflict of interest exists.
